# Newborn Screening for Sickle Cell Disease in Europe

**DOI:** 10.3390/ijns5010015

**Published:** 2019-02-12

**Authors:** Yvonne Daniel, Jacques Elion, Bichr Allaf, Catherine Badens, Marelle J. Bouva, Ian Brincat, Elena Cela, Cathy Coppinger, Mariane de Montalembert, Béatrice Gulbis, Joan Henthorn, Olivier Ketelslegers, Corrina McMahon, Allison Streetly, Raffaella Colombatti, Stephan Lobitz

**Affiliations:** 1 Public Health England, NHS Sickle Cell and Thalassemia Screening Programme, London SE16LH, UK; 2 Laboratoire d’Excellence GR-Ex, UMR_S1134, Inserm, Université Paris Diderot, Sorbonne Paris Cité, Institut National de la Transfusion Sanguine, 75015 Paris, France; 3 NBS Laboratory for Haemoglobinopathies, Hôpital Universitaire Robert-Debré, 75019 Paris, France; 4 Département de génétique médicale, Aix-Marseille Université, Hôpital de la Timone, 13385 Marseille, France; 5 National Institute for Public Health and the Environment, Centre for Health Protection, 3720 Bilthoven, The Netherlands; 6 Pediatric Medicine Laboratory, Department of Pathology, Mater Dei Hospital, Triq Tal-Qroqq, MSD2090 Msida, Malta; 7 Department of Pediatric Oncology/Hematology, Hospital Universitario General Gregorio Marañón, Facultad de Medicina, Universidad Complutense Madrid, 28007 Madrid, Spain; 8 Department of Pediatrics, Reference Center for Sickle Cell Disease, AP-HP Hôpital Universitaire Necker-Enfants Malades, 75743 Paris, France; 9 Department of Clinical Chemistry, Cliniques Universitaires de Bruxelles, Hôpital Erasme—ULB, 1070 Bruxelles, Belgium; 10 Laboratoire—Biologie Clinique, Centre Hospitalier Régional de la Citadelle, 4000 Liège, Belgium; 11 Our Lady’s Children’s Hospital, Crumlin, D12V004 Dublin, Ireland; 12 School of Population Health and Environmental Sciences, Faculty of Life Sciences & Medicine, King’s College London, London WC2R2LS, UK; 13 Division of Healthcare Public Health, Health Protection and Medical Directorate, Public Health England, London SE18UG, UK; 14 Department of Child and Maternal Health, Clinic of Pediatric Hematology/Oncology, Azienda Ospedaliera-Università di Padova, 35129 Padova, Italy; 15 Department of Pediatric Oncology/Hematology, Kinderkrankenhaus Amsterdamer Straße, 50735 Cologne, Germany

**Keywords:** screening, sickle cell disease, newborn

## Abstract

The history of newborn screening (NBS) for sickle cell disease (SCD) in Europe goes back almost 40 years. However, most European countries have not established it to date. The European screening map is surprisingly heterogenous. The first countries to introduce sickle cell screening on a national scale were France and England. The French West Indies started to screen their newborns for SCD as early as 1983/84. To this day, all countries of the United Kingdom of Great Britain and Northern Ireland have added SCD as a target disease to their NBS programs. The Netherlands, Spain and Malta also have national programs. Belgium screens regionally in the Brussels and Liège regions, Ireland has been running a pilot for many years that has become quasi-official. However, the Belgian and Irish programs are not publicly funded. Italy and Germany have completed several pilot studies but are still in the preparatory phase of national NBS programs for SCD, although both countries have well-established concepts for metabolic and endocrine disorders. This article will give a brief overview of the situation in Europe and put a focus on the programs of the two pioneers of the continent, England and France.

## 1. England

### 1.1. Historical Background

Newborn screening (NBS) for sickle cell disease (SCD) was implemented in England between 2002 and 2005. Migration to the United Kingdom (UK) after 1945 had led to an increase in the population at risk from SCD, whilst the 1972 USA National Sickle Cell Anemia Control Act [1] had resulted in increased recognition and politicization of the disorder. The 1986 randomized trial of penicillin in SCD children aged less than 5 by Gaston et al. [2] demonstrated improved outcomes and provided a clear justification for NBS for SCD. Pockets of NBS had been implemented locally in some high prevalence areas in the 1970s but these were not systematic and resulted in inequitable policies throughout the country. These factors led to a major review by a Health Technology Assessment [3] working party which formed the basis for subsequent policy decisions. Political commitment was achieved via the National Health Service (NHS) plan in 2000 [4] and this led to the formation of a small team to plan and drive implementation of a linked newborn and antenatal screening program.

At this time, despite political commitment there were no policies and no financial resources had been allocated. Funding was only able to be sourced once an implementation plan had been developed. The working group had to resolve the controversy and sensitivities associated with implementing a genetic test in a minority group along with resistance from some who questioned the value of the program. There was also conflict between the desires of stakeholders and what could realistically be achieved. Initially overseas practices were reviewed, and a survey of local practices was carried out to inform decisions. A steering group was established in 2001, comprised of key health professionals, users and representatives from patient groups to endorse the policy decisions put forward by the expert working sub groups. Sub groups were developed to work on specific issues such as training, laboratory practices, education and information for users and professionals. The UK National Screening Committee makes recommendations to ministers about all decisions regarding screening policy in the UK and in 2002 approval was given for universal screening of SCD in children. The techniques in use also detected other hemoglobinopathies including beta thalassemia disease (defined clinically as transfusion dependent beta thalassemia or non-transfusion dependent beta thalassemia or HbE/beta thalassemia) thus it was also agreed that these cases along with all other hemoglobin variants detected would be reported. Policy was subsequently updated following review in 2011 such that the only other haemoglobins now reported are beta thalassemia disease, hemoglobins S, C, D, E and O^Arab^, along with any variant co-inherited with Hb S. The four countries which form the UK operate semi-autonomously and screening in Scotland, Northern Ireland and Wales was adopted later. Scotland and Northern Ireland broadly follow the English policy whilst Wales implemented screening much later and has its own strategy [5].

### 1.2. Implementation

Along with stakeholder engagement, initial work was related to the development of policies for implementation. These covered areas such as sample type, technology to be used, testing protocols, reporting, delivery of results and referral into care. It was agreed that the blood spot sample already collected for metabolic screening would be used and that the program would be incorporated into the newborn blood spot screening pathway. Following reorganization there were 13 laboratories performing NBS in England, and thus implementation required policies which ensured consistency as well as a significant training investment. A two-stage testing protocol was implemented to increase the robustness of data with only high-performance liquid chromatography (HPLC) and iso-electric focusing (IEF) recognized as acceptable techniques (https://www.gov.uk/government/publications/sickle-cell-and-thalassemia-screening-handbook-for-laboratories). Counselling was aligned with genetics and different models were developed to fit with local prevalence of the conditions, resources and practices. Areas with high incidence had dedicated counsellors. Clinical policies and standards were developed, and these were subsequently published (https://www.gov.uk/government/publications/sickle-cell-disease-in-children-standards-for-clinical-care). Some funds were provided towards the development of initiatives such as transcranial Doppler scanning. Standards and the key performance indicators to measure performance against these standards were introduced (https://www.gov.uk/government/publications/standards-for-sickle-cell-and-thalassaemia-screening). The standards have evolved over time and are now reviewed annually to ensure they remain fit for purpose. A communication strategy is an important aspect to ensure engagement in the process. Educational materials such as booklets and leaflets for professionals, parents and the public (available in different languages) were developed. More recently blogs and other online resources have been used. Information and educational resources were developed for professionals and a variety of education and training for NHS screening staff is available (https://www.gov.uk/guidance/sickle-cell-and-thalassaemia-screening-education-and-training#haemoglobinopathies-sct-screening-programme-update).

Annual data collection and reporting has been developed and the information is used to inform decisions and to monitor trends, performance and the effect of changes in practice. Quality assurance of the whole pathway via peer review and inspection has been an important factor in ensuring consistency and maintaining standards. The ongoing evaluation present since the program’s inception has led to the development of initiatives such as a failsafe to ensure samples are received on all babies and provision for the testing of babies who have been transfused. An initiative to measure outcomes from early detection of SCD in England was commenced in 2010 and has subsequently reported [6].

### 1.3. Results

More than 8 million babies have now been screened in the England; 3686 screen positive babies were identified between 2005 and 31 March 2017 [6], a summary of results is shown in Table 1.

The recently published newborn outcomes project which assessed timeliness of entry into care and treatment, antenatal screening history, and mortality reported on 1313 screen positive infants [6]. The paper concludes that the screening program accurately identifies babies with SCD, enrolment into care is timely but does not meet program standards, and despite mortality being low deaths still occur from invasive pneumococcal disease with adherence to antibiotics remaining important.

### 1.4. Summary

The long road from the first pockets of screening to full implementation of universal NBS for SCD in the whole of the UK took almost 30 years. Initially implementation was in England followed by Scotland, Northern Ireland and eventually Wales in 2014. Implementation was driven by increasing immigration and public pressure for inclusion in health care strategy. Testing for SCD is now firmly embedded in newborn blood spot screening which is assessed as a complete pathway rather than individual components. Additionally as part of linked antenatal and newborn screening, the program acts as a quality measure for antenatal screening with any apparent discrepancies being investigated. A key element in the success of the program has been the input of the small team who have driven the process, and ensured and maintained stakeholder engagement and development and implementation of policies. Ongoing evaluation and assessment is an essential aspect to ensure the program is fit for purpose. The program has proven itself to be adaptable, for example by adopting new technologies such as mass spectrometry testing [7,8]. The use of the new technology to withhold detection of carrier conditions has led to differing strategies in the UK. Within England, consistency has been maintained despite some adoption of this technique. Balancing all stakeholder expectations and requirements continues to be a challenge in the program.

The newborn outcomes project has shown that the program accurately identifies babies with SCD but there are some issues including acceptance of penicillin and delays between babies’ positive screening results and enrolment into care and mortality associated with invasive pneumococcal disease. A detailed review into all aspects of the process and stakeholder requirements (a discovery process), identified a lack of a centralized view of the baby’s progress along the pathway, lack of feedback to data providers, and duplication of data entry. A new improved system has been developed which will:

1. Automate the process of referring screen-positive babies into treatment centres and record their health outcomes;
2. Help ensure affected babies are treated as early as possible;
3. Provide oversight of the progress along the pathway to prevent babies getting ‘lost’ in the system;
4. Improve the process of collecting and sharing data;
5. Remove the need for clinicians to enter the same data twice;
6. Integrate with the National Hemoglobinopathy Register and National Congenital Anomalies and Rare Disorders Registration Service.

Beta thalassemia continues to be reported when found in NBS for SCD, and this has been due to lack of available evidence on the efficacy of detection and the clinical justification for early detection. There is now improved evidence on the sensitivity and specificity of the test [9,10]. However published clinical evidence remains scarce.

In conclusion, over 10 years’ experience in England has shown this to be a robust program, which benefits from technical expertise along with strong stakeholder engagement. A key aspect of the program’s success has been the development of clinical care pathways to ensure screen positive babies receive appropriate care in a timely manner.

## 2. France

### 2.1. Historical Background and Implementation

The first pilot study on NBS for SCD in France was conducted on the Caribbean island of Guadeloupe in 1983 and led to a universal screening program in Guadeloupe within one year [11]. The program was rapidly extended to Martinique, and in 1986 the first pilot studies in Metropolitan France (Paris and Marseille) were started.

In 1995, SCD became an additional target disease of the national NBS program in France (together with phenylketonuria, congenital hypothyroidism, congenital adrenal hyperplasia and cystic fibrosis). However, although the French NBS is universal in nature, screening for SCD in Metropolitan France is targeted at couples at risk to date, while in French overseas territories SCD screening is universal [12].

NBS for SCD is centralized in France and performed in five reference laboratories. Three of these are located in Metropolitan France (Paris, Marseille, Lille) and two are run on the Caribbean islands of Guadeloupe and Martinique. Since the year 2000, all French maternity clinics (public and private) are covered by the program which receives funding from the French social security system.

### 2.2. Methods

NBS for SCD in France is done in a two-tier strategy from heel prick dried blood spots. Initially, IEF was the method of choice. Today, the first-tier methods are HPLC, capillary electrophoresis (CE) and very recently MALDI-TOF mass spectrometry found its way into NBS in one laboratory. Second-tier testing is done with an established method different from the first one (e.g., first-tier: HPLC; second-tier: CE). The United Kingdom National External Quality Assessment Service (UK NEQAS) provides the external quality control assessment for the French NBS program. Absolute numbers of new cases, incidence, coverage and genotypes are collected and published annually globally and for each of the French regions (http://www.afdphe.org/sites/default/files/bilan_afdphe_2016.pdf). Screening results consistent with SCD are communicated to the families by a trained pediatrician of the national care program. Parents of carriers are informed by mail and offered information by a physician or a genetic counsellor. Testing of the father is recommended to identify couples at risk of having a significant disorder of hemoglobin. In this latter context, genetic counselling including extended information on SCD is provided and the opportunities of having prenatal or preimplantation genetic diagnosis are mentioned. A psychologist will participate in the process whenever necessary.

### 2.3. Results

Between the beginning of the national program in 1995 and 2016, 5.4 million newborns have been screened and 7,644 of tested newborns were diagnosed with SCD. A summary of results is shown in Table 2.

In 2016, 431 cases of SCD were identified by NBS which corresponded with a birth prevalence of 1 in 1836, i.e., the highest among all target diseases tested in the French national NBS program. 356 of these 431 cases were diagnosed in Metropolitan France, and 218 of the 356 cases in Metropolitan France were diagnosed in the Ile de France, i.e., in the Parisian area. This translated into a birth prevalence of 1 in 824 in the Ile de France. In the French Overseas, 75 babies were affected by SCD. This was a mean birth prevalence of 1 in 525, varying from 1 in 1968 on the Island of Réunion and 1 in 206 in French Guiana (http://www.afdphe.org/sites/default/files/bilan_afdphe_2016.pdf).

The birth coverage by universal NBS in the French Overseas is over 98%. In the targeted program of Metropolitan France, a mean of 38.9% of all newborns was screened in 2016 (minimum: 9.1% in the Brittany region; maximum: 73.6% in the Ile de France). Only 277 parents (0.035%) refused to participate to the French NBS program in 2016. Between 2006 and 2016, there was a significant increase of couples at risk that led to a subsequent increase of the newborns screened in the targeted program in Metropolitan France from 27 to 38.9% (see above). In the same period, the prevalence of affected newborns rose by 24%.

The targeted SCD NBS in Metropolitan France is probably more cost-effective than a universal approach. The present cost per test is EUR 3.06. This equals to total costs of EUR 1.02 million per year for the whole program or EUR 2,359 per newborn identified. However, there are several drawbacks: most importantly, about 2% of affected newborns are missed, because they are not screened for various reasons [13]. Hence, patients’ associations and most of the concerned medical health care professionals advocate a universal screening approach. Beyond missed cases, arguments in favor of universal screening include putative discrimination and inequality to the access to care. Still, in its December 2013 orientation report the *Haute Autorité de Santé* concluded that there is currently no evidence to justify universal NBS for SCD in Metropolitan France.

### 2.4. Follow-Up and Organization of Care

The first French Sickle Cell Disease Comprehensive Care Center was established in Guadeloupe in 1990 [11]. In 2005, a pioneering national program on rare diseases was started in France. Its objectives include the definition of pathways to access to care, the prevention of diagnostic wavering, the establishment of reference centers, the organization of specialized training for health care professionals, the development of information platforms for patients, relatives, health care professionals and the community, and the promotion of research.

During the first phase of this program (2005–2008), two accredited reference centers for SCD (Ile de France and French Antilles), one reference center for thalassemia (Marseille) and a network of reference laboratories were founded. The mission of these institutions was to establish clear healthcare channels for the patients through the formation of a national network of qualified centers. In 2005, national treatment guidelines for SCD were published online.

In the second phase of the national program for rare diseases (2011–2014), 13 constitutive reference centers and 46 competence centers in addition to the coordination reference centers were established to ensure a dense and comprehensive coverage of the whole French national territory.

A national registry for thalassemias has been established that included 692 patients (beta thalassemia: 612; alpha thalassemia: 80) in 2016, but a national registry for SCD is only now being established. (Definitions: beta thalassemia = transfusion dependent beta thalassemia or non-transfusion dependent beta thalassemia or HbE/beta thalassemia; alpha thalassemia = alpha thalassemia hydrops fetalis syndrome, HbH disease and HbH-Constant Spring disease.)

The whole network was certified in 2017 for five years. The program is evaluated regularly for its efficiency by health authorities and through published studies [14,15] and the next turn of certification is to occur in 2022.

In addition, local networks have been established at the initiative of some dedicated physicians, like RoFSED (*Réseau Francilien de Soin des Enfants Drépanocytaires*) in the Parisian area, and supported by public funds to connect the national health care system with proximity stakeholders, e.g., general practitioners, local health care and social professionals and school personnel. They are essential actors in terms of therapeutic education of the patients and parents and information of the general public (e.g., brochures, educational sessions and tools, games, films etc.; for examples, please see: http://www.rofsed.fr).

## 3. The Netherlands

The Netherlands has had a national NBS since 1974. Participation is voluntary, but virtually all of the newborns (approximately 175,000 annually) are tested. Since 2015 also newborns from the Dutch Caribbean, Bonaire, St Eustatius and Saba (200–250 children), are screened. For this, the specimens are sent to one of the five designated NBS laboratories in The Netherlands. Specimens are heel prick dried blood spots that are currently examined for 19 conditions. 16 of which are endocrine and metabolic disorders. The other three are blood disorders. NBS for SCD was introduced in 2007 [16,17] and in 2017 the program was extended to include beta thalassemia major and hemoglobin H disease [18,19].

An inquiry performed in 2002 resulted in the estimation of 50 sickle cell patients born annually and at least 800 SCD patients in total [20]. The total number of SCD patients, adults and children, in The Netherlands is now estimated to be in an order of 1500–2000. This number is based on expert opinion only, but clinicians and researchers are working together to set up a national registry.

NBS for hemoglobinopathies is universal, i.e., without any preselection on the basis of the putative ethnic origin of the baby. The method of choice to analyze dried blood spots is HPLC. A distinctive feature of the Dutch program is that there is no second-tier method in place. Children with positive screening tests are referred to pediatric hematologists for confirmation. In spite of this practice, only five false positives were reported in the first 10 years of screening. Almost all of these false positives were carriers of HbS or had another hemoglobinopathy. The first year of screening resulted in 40 diagnoses of SCD [21]. The 2016 annual bulletin reported 30 referrals for SCD and 30 referrals for alpha and beta thalassemia combined (https://www.rivm.nl/sites/default/files/2018-11/HielprikMon2016-Engels-def.pdf). In addition 800–850 carriers are detected every year [22]. The carrier state is reported routinely to the general practitioner to facilitate family testing if parents do not object to knowing the carrier status of their child.

## 4. Spain

A national universal newborn screening program has been available in Spain since 1978. It is on a voluntary basis, but has a nearly 100% coverage. Universal screening for hemoglobinopathies started in the Madrid region in 2003, in the Basque country in 2011, in Valencia in 2012, and was extended nationwide in 2015. More than 400,000 neonates were screened in 2016 with 48 cases of SCD identified [23]. HPLC is the first test used in most of the regions. Confirmatory testing is done with HPLC on the same dried blood spot and on a new capillary sample before the age of three months.

## 5. Belgium

Belgium does not screen for hemoglobinopathies on a national level. However, there are currently three regional programs in place [24]. The *Cliniques Universitaires de Bruxelles, hôpital Erasme* (currently the LHUB-ULB) are responsible for the Brussels region. They were the first institutions to introduce NBS for SCD in Belgium in 1994 [25,26,27,28] and the only institutions to receive public funding until 2015.

Both of the other two screening projects are located in Liège at the *Centre Hospitalier Régional de la Citadelle* (CHR Citadelle), which started NBS for SCD solely in its own institution in 2002 [27] and the *Centre Hospitalier Universitaire de Liège* (CHU de Liège) that covers 14 maternity wards in Wallonia since 2007 [29,30]. SCD screening in the Liège region has a quasi-official status, but is not reimbursed by the insurance companies. It is financed through hospital funding.

Since 2008, Belgium has been running a registry for SCD patients. From the academic point-of-view it is interesting that all patients diagnosed with SCD in Belgium are reported to this registry. While some of them have been identified through NBS, others have been identified later in life on the basis of clinical symptoms. By comparing the two populations, the Belgian group was able to demonstrate that NBS for SCD improves clinical outcome by a significant reduction of bacteremia and hospitalization [31,32].

To complement the screening data from the well-established programs in Brussels and Liège, two thirds (n = 39,599) of all Belgian newborns were screened in a six-month pilot study [24]. The highest incidences were found in urban areas. However, in total, SCD was more common than any other target disease in the Belgian NBS program. 17 babies (1:2329) were diagnosed with sickling disorders. 1 in 77 was demonstrated to be heterozygous for hemoglobin S.

In terms of the methodology, the Belgian programs are heterogenous. While LHUB-ULB and CHR Citadelle used to screen by CE from liquid cord blood, CHU de Liège applied tandem mass spectrometry to dried blood spots on heel prick filter paper. HPLC and molecular genetics are used as confirmatory methods. In the pilot study, dried blood spots were examined by capillary electrophoresis. Positive results were confirmed by either HPLC or IEF. All Belgian projects have in common that all newborns were screened without any preselection on the basis of their putative ethnic origin (i.e., universal screening).

## 6. Italy

A National Newborn Screening Program (NBS) has been present in Italy since the nineties and around 500,000 babies are born every year. The National Health System is organized on a regional basis and, therefore, every region can organize its own NBS. Therefore, there are significant differences in the regional legislations regulating NBS and in the development of the regional NBS programs in terms of the diseases to be included, funding allocated and program organization (http://www.aismme.org/images/Linee_guida_05.08.pdf). The screening is mandatory but does not include hemoglobinopathies. Nevertheless, since the end of 2000, due to the increase of patients with SCD as a result of immigration, several scientific societies have started to raise the issue of NBS for SCD: the Italian Association of Pediatric Hematology Oncology (AIEOP) highly recommended NBS for SCD in its guidelines published in 2012 in Italian and 2013 in English [33] and in 2017 the AIEOP together with the Italian Society of Thalassemia and Hemoglobinopathies (SITE) issued a joint document for the recommendation of NBS for SCD in Italy (http://www.site-italia.org/file/collana_scientifica/libretto_5_2017/index.php). These efforts have led to the development of six NBS pilot programs [34,35,36,37,38,39], three of which are currently active [35,38,39] and one is being scaled up [37]. Two programs are universal [37,39] and the remaining are targeted [35]; one is antenatal. Methodologies and techniques vary: some use cord blood, others dried blood spot, HPLC is usually the first test whilst the confirmatory test can be molecular biology or IEF. Overall, the results of the universal programs are in line with those of other European countries with high immigration, with 1.16% incidence of positive tests (0.07% with SCD, 0.68% with sickle cell trait) [37].

## 7. Germany

NBS in Germany started in 1969 with nationwide testing of all newborns for phenylketonuria. Over the years, the number of targeted diseases rose to 16 with cystic fibrosis and tyrosinemia being the last implemented in 2016 and 2017, respectively. All target diseases are endocrine or metabolic disorders. NBS in Germany is offered to every family, but participation is voluntary. However, coverage is very close to 100%. In 2015, 737,575 babies were born in Germany and 539 confirmed diagnoses of NBS target diseases were made (1:1368).

SCD is not a target disease of the German NBS program. Principally, this is owing to the fact that the disease has not been endemic in Germany in the past. It has thus been neglected over decades, although a significant and steady increase of diagnoses was observed by Kohne and Kleihauer as early as in the late 1960s [40,41]. The first systematic attempt to identify newborns suffering from SCD was made by Genzel who started a targeted pilot project in her Department of Obstetrics in Munich [42]. One affected baby was diagnosed in a targeted screening approach of 306 children born to Sub-Saharan African mothers.

With the formation of the *GPOH-Konsortium Sichelzellkrankheit* and its official assignment by the German Society for Pediatric Oncology and Hematology (GPOH) to develop a management program for children and adolescents with SCD, awareness and care improved dramatically. To date, members of this consortium have completed four regional pilot studies on NBS for SCD [43,44,45,46,47]. Two have been done in the Berlin NBS laboratory and one in the Hamburg and the Heidelberg NBS laboratory, respectively. Another study in Berlin is currently recruiting patients and a joint German–Polish pilot project has recently been started.

Altogether, dried blood spot samples of 118,019 newborns have been investigated in the four published studies and 32 affected babies (1:3688) were identified as having SCD. These results led the authors to the conclusion that NBS for SCD is clearly indicated in Germany.

The formal application to introduce NBS for SCD was assessed by the *Gemeinsamer Bundesausschuss* (GBA) in May 2018 for the first time. The GBA is the political body that decides what the public health insurance companies have to pay for. It consists of representatives of the different interest groups in the German healthcare system (e.g., patients, doctors, insurance companies etc.). The GBA has admitted the application for the highly formal decision-making process. A result is not expected before May 2021.

## 8. Ireland

Ireland has had a national NBS program since 1966, for metabolic and endocrine diseases using heel prick and dried blood spot analysis. Participation is mandatory but opting out is possible. Currently the program captures 99.9% of all newborn infants. Targeted and voluntary national hemoglobinopathy screening has been available since 2003. Currently, it is performed on cord blood using CE as first line testing and HPLC/IEF as a confirmatory test [48]. The number of newborns diagnosed by NBS with SCD or beta thalassemia major is 10–15/year and all are referred to a single pediatric hemoglobinopathy center.

## 9. Malta

In Malta, all newborn are screened for SCD (universal NBS program). In 2017, 4370 babies were tested, none of which suffered from the disorder. However, eight carriers were detected. As in many other countries, a two-tier strategy is applied. IEF serves as the first tier method. Any sample containing Hb S (i.e., heterozygous, homozygous or compound heterozygous, respectively) is re-examined by HPLC to confirm the first-tier result. In case of a positive result, the screening laboratory notifies the thalassemia and sickle cell clinic who in turn will notify the parents and an appointment is given for a follow up. If requested, parents and siblings are screened (personal communication).

## 10. Closing Remarks

Despite all these varieties, there is broad consensus among European NBS and SCD experts on the fundamental standards that a state-of-the-art NBS program for SCD should fulfil. These standards have recently been defined in a two-day consensus conference of more than 50 experts from 13 European countries and published elsewhere [49,50]. Future intensification of this collaboration will hopefully lead to a harmonization of NBS programs for SCD in Europe.

## Figures and Tables

**Table 1:** Screen positive babies between 2005 and 31 March 2017.

Screening Result	Number	Percentage
FS	2580	70.0%
FSC	927	25.1%
FS + other hemoglobin variant *	179	4.9%

* Refers to Hb D, E, O^Arab^ and any other rare variants detected.

**Table 2:** Screen positive babies between 1995 and 2016.

Screening Result	Number	Percentage
FS	6015	78.7%
FSC	1600	20.9%
FS + other hemoglobin variant *	29	0.4%

* Refers to Hb D, E, O^Arab^ and any other rare variants detected.
